# In Search of the Hyperglycemic Threshold Required to Induce Growth Hormone (GH) Suppression

**DOI:** 10.7759/cureus.34463

**Published:** 2023-01-31

**Authors:** Maria João Bugalho, Mariana Lopes-Pinto, Carlos Lemos, Ema Nobre

**Affiliations:** 1 Serviço de Endocrinologia, Centro Hospitalar Universitário Lisboa Norte, Hospital de Santa Maria, Lisboa, PRT; 2 Clínica Universitária de Endocrinologia, Faculdade de Medicina, Universidade de Lisboa, Lisboa, PRT; 3 Serviço de Patologia Clínica, Centro Hospitalar Universitário Lisboa Norte, Hospital de Santa Maria, Lisboa, PRT; 4 Medicina Laboratorial, Faculdade de Medicina, Universidade de Lisboa, Lisboa, PRT

**Keywords:** oral glucose tolerance test, suppression test, growth hormone, acromegaly, hyperglycemia

## Abstract

Introduction

According to the 2014 Endocrine Society Clinical Practice Guideline on acromegaly, the confirmation of acromegaly diagnosis is established by finding a lack of suppression of growth hormone (GH) to < 1 ug/L following documented hyperglycemia during an oral glucose tolerance test. However, in this setting, the concept of hyperglycemia has never been clearly defined.

Objective

This study aimed to define the hyperglycemic threshold required to induce GH suppression.

Methods

We retrieved the glycemia profile of 44 individuals after a standard 2-h 75g oral glucose tolerance test prescribed to assess GH suppression and performed a comprehensive analysis of two subgroups of individuals (28 reaching GH suppression and 16 in whom GH suppression was not observed). All of the data were analyzed with the program Graph Pad Prism. Differences between means were assessed by Student’s unpaired t-test or Mann-Whitney U test as deemed appropriate. Fisher’s exact test was used for categorical variables.

Results

Individuals in G1 and G2 were different only for the median basal GH and median IGF-1.

No significant differences in terms of the prevalence of diabetes and prediabetes were found. The glucose peak was achieved earlier in the group that reached GH suppression.

The median of the highest glucose values of both subgroups was not different. A correlation between peak and baseline glucose value was found only among those in whom GH suppression was reached. Among these, the median glucose peak (P50) was 177 mg/dl, whereas the 75^th^ percentile (P75) and 25^th^ percentile (P25) were 199 mg/dl and 120 mg/dl, respectively.

Conclusion

Considering that 75% of those in whom GH suppression was observed after an oral glucose overload test reached blood glucose values above 120 mg/dl, we propose to use this value as the blood glucose threshold for inducing GH suppression. In light of our results, whenever GH suppression is not observed; and the highest glycemic value is below 120 mg/dl, it might be useful to repeat the test prior to any conclusion.

## Introduction

Acromegaly is a disorder caused by chronic growth hormone (GH) hypersecretion leading to IGF-1 over-production. Screening of this condition is performed by an IGF-1 test since GH is normally released in pulses and random levels vary widely. Investigation of GH suppression during an Oral Glucose Tolerance Test (OGTT) 75 g for two hours is recommended as a confirmatory test for those cases with elevated age- and sex-adjusted IGF-1 levels. In acromegaly, suppression failure occurs, and there may be a paradoxical rise in GH in response to the glucose challenge [1].

Recommendations propose confirmation of the diagnosis by finding a lack of suppression of GH to < 1 ug/L following documented hyperglycemia during an OGTT [2].

In a known acromegalic, cure reassessment after pituitary surgery may be indicated for those with a random GH at 12 weeks or later greater than 1 µg/L. The value to define disease control is less well established, but cut-offs of 1 ug/L and 0,4 ug/L have been suggested [3,4].

Recent evidence suggests that GH nadir concentrations, as measured by a modern GH assay, are much lower than the current cut-offs mentioned in guidelines for the diagnosis and follow-up of acromegaly [5]. Moreover, several studies have shown that patients with mild acromegaly may have nadir GH lower than 1 μg/L [6-8].

Independently of the GH nadir considered, hyperglycemia is the necessary condition to reach GH suppression. A consensus has never been achieved in defining at which glycemic threshold occurs GH suppression.

Aiming to address this issue, we reviewed the results of OGTT tests performed from 2016 to 2021 and analyzed the glycemic profile of those individuals in whom GH suppression was reached.

## Materials and methods

We retrieved all GH suppression tests performed in the period encompassing the years 2016 to 2021. In parallel, the IGF-1 levels preceding the tests were also collected.

Standard 2-h 75g OGTT was performed in each subject after overnight fasting. Blood was drawn through an indwelling venous cannula at -15, 0, 30, 60, 90, and 120 minutes, after oral glucose administration, for plasma glucose and GH levels. The patients remained at rest throughout the test. The diagnosis of diabetes or pre-diabetes was not known before OGTT was performed.

Basal glucose and GH were defined as the mean of values registered at -15 minutes and 0 corresponding the latter to the time immediately before glucose administration.

Tests in whom the glucose load elicited a paradoxical increase in GH were excluded.

Impaired fasting glucose is defined as fasting plasma glucose of 100-125 mg/dL. Impaired glucose tolerance is defined as 2-h plasma glucose 140-199 mg/dL after OGTT. Diabetes Mellitus was defined as having either fasting plasma glucose ≥ 126 mg/dL or 2-h OGTT plasma glucose ≥ 200 mg/dL.

Serum GH was measured by an electrochemiluminescence immunoassay (ROCHE - Elecsys hGH, lower detection limit 0,030 ng/ml, according to the manufacturer's indications). 

IGF-1 was measured by an immunoassay (ROCHE - Elecsys IGF-1, lower detection limit 7 ng/ml, highest measure value without dilution 1600 ng/ml, according to the manufacturer's indications).

Tests were divided into 2 subgroups: G1 - GH nadir more than 1 ug/L following oral glucose administration and G2 - GH nadir less than 1 ug/L following an oral glucose load. Thereafter, glycemia percentiles of individuals within this latter group were determined.

All of the data were analyzed with the program Graph Pad Prism version 9.3.1 (GraphPad Software). Quantitative variables were tested for Gaussian distribution with the Shapiro-Wilk test. Differences between means were assessed by Student’s unpaired t-test or Mann-Whitney U test as deemed appropriate. Fisher’s exact test was used for categorical variables. Receiver Operator Characteristic (ROC) analysis was used to evaluate the diagnostic performance of glycemia as a predictor of the outcome in terms of GH suppression. The area under the ROC curve (AUC) was used to assess overall diagnostic accuracy.

A value of p < 0.05 was accepted as denoting statistical significance.

## Results

We identified 46 tests. Two tests in which a paradoxical increase was observed were excluded since we were searching for the necessary glycemic threshold to induce GH suppression.

G1 included 16 tests performed on 10 women and 6 men. G2 included 28 tests performed on 20 women and 8 men. The mean age in G1 and G2 was 50±18.6 and 54±12 years, respectively.

The review of clinical files of individuals in each group allowed the integration of clinical, laboratory, and semiological data conducting to the conclusion that all but one patient in G1 had a diagnosis of acromegaly (10 already submitted to surgery, five awaiting surgery); the exception was a patient with a macro prolactinoma submitted to the suppression test because of an abnormal basal GH despite a normal IGF-1.

G2 included 14 patients with past surgery for acromegaly and 14 individuals in whom the clinical and laboratory diagnosis of acromegaly was doubtful.

Individuals in G1 and G2 were different only for the median basal GH (4.83 ng/ml versus 1 ng/ml) and median IGF-1 (300 ng/ml versus 227 ng/ml).

As summarized in Table 1, the median glucose peak was not significantly different between groups. Moreover, a ROC analysis revealed an AUC of 0.5859.

**Table 1:** Comparative analysis of G1 and G2. ns: not statistically significant

	G1 (GH nadir >1 ug/L n = 16	G2(GH nadir < 1 ug/L n = 28	p
Gender			ns
Female	10	20	
Male	6	8	
Mean Age (years)	50±18.6	54±12	ns
Baseline Glucose (mg/dl/mmol/L)			
Mean	95±12.8/5.3±0.71	95±15/5.3±0.83	ns
Minimum	78/4.3	63/3.5	
Maximum	113/6.3	130/7.2	
Glucose peak after 75 g oral glucose (mg/dl/mmol/L)			
Median	184	177	ns
Ratio between the glucose peak and the baseline value			
Mean	2.03±0.43	1.9±0.49	ns
Minimum	1.57	1.01	
Maximum	2.88	3.2	
Oral Glucose Test			ns
Diabetes	3	4	
Pre Diabetes	6	8	
Baseline GH (ng/ml = ug/L)			
Median	4.83	1.05	0.0001
IGF1 (ng/ml/nmol/L)			
Median	300/29.74	227/39.3	0.0072

No significant differences in terms of the prevalence of diabetes and prediabetes were found. Prediabetes was observed in 37,5% and 28,6% in G1 and G2, respectively, and diabetes mellitus in 18,8% and 14,3%, respectively.

**Figure 1:**
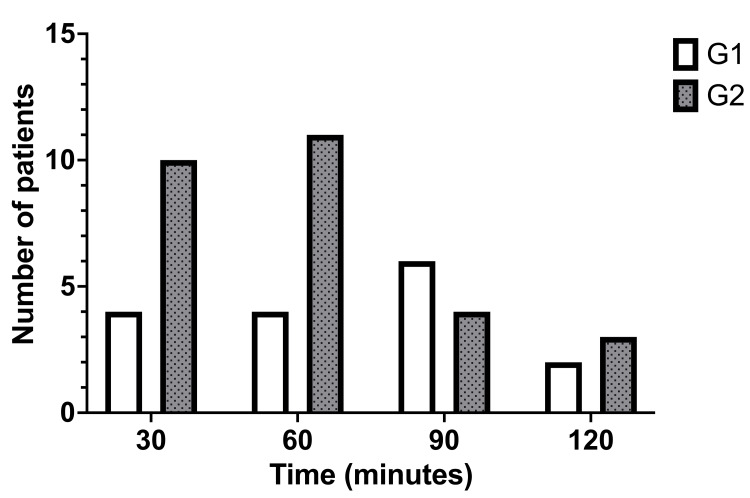
Time to glucose peak.

The glucose peak was achieved earlier in group G2 (Figure 1): 75% of the patients reached the highest value at or < 60 minutes.

**Figure 2:**
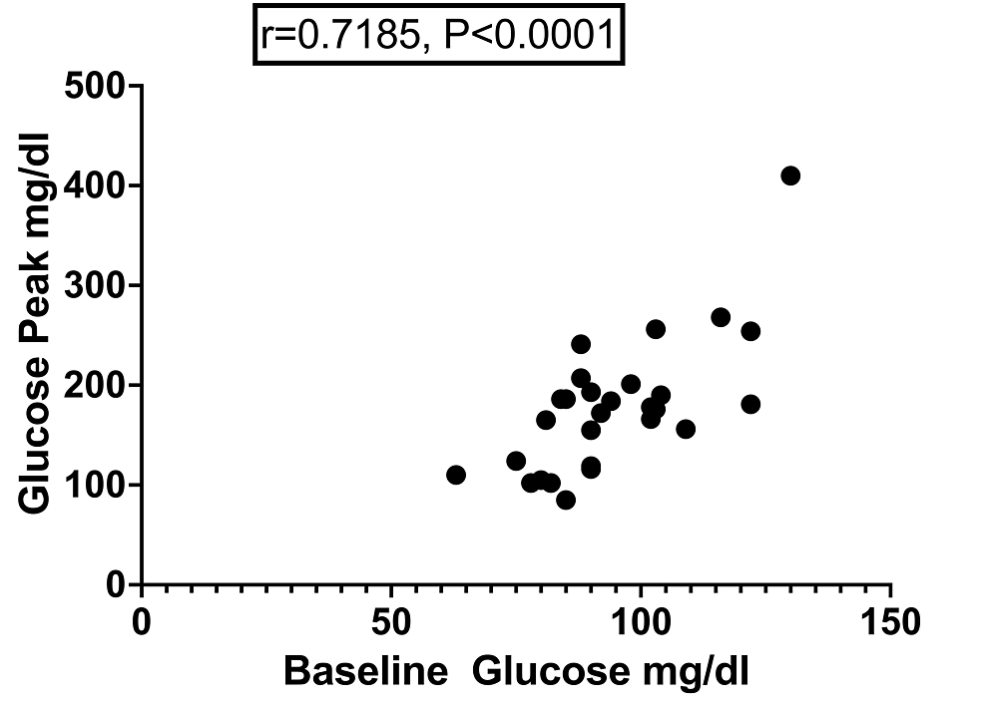
Correlation between glucose peak and baseline glucose observed in G2.

Moreover, only in G2 a correlation (r=0.7185, P<0.0001) between the highest value and the basal value of glucose was observed (Figure 2).

Among individuals that reached GH suppression, the median glucose value (P50) was 177 mg/dl (9.8 mmol/L), whereas the 75^th^ percentile (P75) and 25^th^ percentile (P25) were 199 mg/dl (11 mmol/L) and 120.25 mg/dl (6.7 mmol/L), respectively.

Based on the suppression test, among the 24 acromegalic patients already submitted to surgery, 14 (58.3%) were considered in remission.

## Discussion

The main metabolic consequence of acromegaly is insulin resistance, which may progress to diabetes mellitus. Alexopoulou O et al. [9] reported a prevalence of impaired fasting glycemia or glucose tolerance of 26% and a prevalence of diabetes mellitus of 28% at diagnosis of acromegaly. In the current study, the prevalence of diabetes mellitus in acromegalic patients was 18,8%, and the prevalence of prediabetes was 37,8%. No difference was found in the prevalence of diabetes and prediabetes among patients with active and inactive acromegaly. However, time to glucose peak occurred in the majority of individuals from G2 (patients in remission of acromegaly or normal individuals) at or before 60 minutes, whereas in patients from G1 (with active acromegaly), tended to occur later. The latter pattern is likely to correspond to insulin resistance [10].

Growth hormone secretion is regulated by two hypothalamic hormones: GH-releasing hormone (GHRH) and somatotropin release-inhibiting factor (SRIF). Ghrelin, a gut-derived peptide, has been considered the third regulator of GH secretion with a stimulatory action. Hyperglycemia suppresses ghrelin [11] and is associated with a somatostatin release into the hypophyseal portal blood suppressing GH levels [5].

Oral glucose administration is the standard method for assessing inhibitory control of GH release. What lacks clarification is the level of glucose that should be reached to confidently conclude that there is no GH suppression.

The median glucose peak was not different between groups with or without GH suppression. However, whereas a correlation between the peak and the baseline value of glucose was documented in those in whom the outcome was GH suppression, it was not observed in those who failed to suppress GH. The variable individual degree of insulin resistance among those in the latter group might have contributed to this difference.

There was concordance between the response to OGTT and clinical data. Except for the case of a patient presenting a pituitary macro adenoma without acromegalic phenotype and high levels of prolactin consistent with the diagnosis of macro prolactinoma. Moreover, the patient had a normal IGF-1 and elevated GH that was not suppressed by hyperglycemia. Although we cannot exclude a mixed somato-lactotroph tumor, alternatively, considering that venepuncture has been regarded as a psychological and physiological stressor on the one hand and on the other that stress can elicit GH secretion, one might speculate to what extent this fact can counteract the inhibitory effect of hyperglycemia.

Glycemia was not a predictor of outcome in terms of GH suppression. The absence of GH suppression observed in G1 was independent of blood glucose, reinforcing the hypothesis that it is an intrinsic feature of autonomous GH secretion.

Furthermore, we observed that the median glucose peak (P50) among individuals who reached GH suppression was 177 mg/dl, whereas the 75^th^ percentile (P75) and 25^th^ percentile (P25) were 199 mg/dl and 120 mg/dl, respectively. Based on this observation and taking into account that 126 mg/dl is the fasting glycemic value accepted to establish the diagnosis of diabetes mellitus, we consider that the value of 120 mg/dl accomplishes the condition of hyperglycemia and may be proposed as the threshold for GH suppression. Further studies involving a larger number of cases are desirable to corroborate current results.

The exact mechanisms underlying GH suppression are not deeply understood. A nadir GH concentration below 1 ug/L was observed in normal individuals either during an OGTT test or saline infusion if GH secretion was evaluated over 180 minutes [12]. Thus, the observation of a GH nadir below 1 ug/L among normal individuals may result from a spontaneous variability and not as a consequence of a glucose overload. On the other hand, other mechanisms, including the release of neuropeptides associated with food ingestion, cannot be ruled out as potential contributors to GH suppression, justifying that in some cases, GH suppression may occur for values below 120 mg/dl as observed in a quarter of the cases of the present series.

## Conclusions

Failure of suppression following documented hyperglycemia has been regarded as diagnostic for acromegaly. However, the term hyperglycemia has never been accurately defined. As a consequence, the interpretation of GH suppression tests is not always straightforward because there are doubts as to whether the theoretical assumption of hyperglycemia has been reached.

In the present study, 75% of the patients in whom GH suppression was achieved had a maximum glycemic value of 120 mg/dl or more. Only in a minority GH suppression was observed despite a maximum blood glucose value of less than 120 mg/dl. Therefore, we propose to use this glycemic threshold to evaluate GH suppression following an oral glucose tolerance test. In light of our results, whenever GH suppression is not observed; and the highest glycemic value is below 120 mg/dl, it might be useful to repeat the test before any conclusion. Due to the small number of cases included, further studies are needed to confirm our findings.

## References

[1] Scaroni C, Albiger N, Daniele A (2019). Paradoxical GH increase during OGTT is associated with first-generation somatostatin analog responsiveness in acromegaly. J Clin Endocrinol Metab.

[2] Katznelson L, Laws ER Jr, Melmed S, Molitch ME, Murad MH, Utz A, Wass JA (2014). Acromegaly: an endocrine society clinical practice guideline. J Clin Endocrinol Metab.

[3] Giustina A, Chanson P, Bronstein MD (2010). A consensus on criteria for cure of acromegaly. J Clin Endocrinol Metab.

[4] Melmed S, Bronstein MD, Chanson P (2018). A consensus statement on acromegaly therapeutic outcomes. Nat Rev Endocrinol.

[5] Hage M, Kamenický P, Chanson P (2019). Growth hormone response to oral glucose load: from normal to pathological conditions. Neuroendocrinology.

[6] Dimaraki EV, Jaffe CA, DeMott-Friberg R, Chandler WF & Barkan AL (2002). Acromegaly with apparently normal GH secretion: implications for diagnosis and follow-up. J Clin Endocrinol.

[7] Ribeiro-Oliveira A Jr, Faje AT, Barkan AL (2011). Limited utility of oral glucose tolerance test in biochemically active acromegaly. Eur J Endocrinol.

[8] Zahr R, Fleseriu M (2018). Updates in diagnosis and treatment of acromegaly. Eur Endocrinol.

[9] Alexopoulou O, Bex M, Kamenicky P, Mvoula AB, Chanson P, Maiter D (2014). Prevalence and risk factors of impaired glucose tolerance and diabetes mellitus at diagnosis of acromegaly: a study in 148 patients. Pituitary.

[10] Chung ST, Ha J, Onuzuruike AU (2017). Time to glucose peak during an oral glucose tolerance test identifies prediabetes risk. Clin Endocrinol (Oxf).

[11] Nakagawa E, Nagaya N, Okumura H (2002). Hyperglycaemia suppresses the secretion of ghrelin, a novel growth-hormone-releasing peptide: responses to the intravenous and oral administration of glucose. Clin Sci (Lond).

[12] Grottoli S, Razzore P, Gaia D (2003). Three-hour spontaneous GH secretion profile is as reliable as oral glucose tolerance test for the diagnosis of acromegaly. J Endocrinol Invest.

